# Recent Progress in Photothermal, Photodynamic and Sonodynamic Cancer Therapy: Through the cGAS-STING Pathway to Efficacy-Enhancing Strategies

**DOI:** 10.3390/molecules29153704

**Published:** 2024-08-05

**Authors:** Kelan Fang, Huiling Zhang, Qinghong Kong, Yunli Ma, Tianchan Xiong, Tengyao Qin, Sanhua Li, Xinting Zhu

**Affiliations:** 1 Guizhou Provincial College-Based Key Lab for Tumor Prevention and Treatment with Distinctive Medicines, Zunyi Medical University, Zunyi 563000, China; 2 College of Basic Medicine, Zunyi Medical University, Zunyi 563000, China; 3 Department of Medicine and Pharmacy, Shizhen College of Guizhou University of Traditional Chinese Medicine, Guiyang 550000, China

**Keywords:** photothermal therapy, photodynamic therapy, sonodynamic therapy, cGAS-STING, cancer therapy, synergistic therapy, immunotherapy

## Abstract

Photothermal, photodynamic and sonodynamic cancer therapies offer opportunities for precise tumor ablation and reduce side effects. The cyclic guanylate adenylate synthase-stimulator of interferon genes (cGAS-STING) pathway has been considered a potential target to stimulate the immune system in patients and achieve a sustained immune response. Combining photothermal, photodynamic and sonodynamic therapies with cGAS-STING agonists represents a newly developed cancer treatment demonstrating noticeable innovation in its impact on the immune system. Recent reviews have concentrated on diverse materials and their function in cancer therapy. In this review, we focus on the molecular mechanism of photothermal, photodynamic and sonodynamic cancer therapies and the connected role of cGAS-STING agonists in treating cancer.

## 1. Introduction

Photothermal, photodynamic and sonodynamic cancer therapies are physical strategies that have demonstrated prominent anticancer efficacy [1,2,3]. These cancer therapy methods integrate light, thermal and acoustic modalities in a single platform to reinforce their therapeutic effects. Photothermal therapy (PTT) uses photothermal agents (PTAs) to convert light into heat. The heat generated by PTAs can induce hyperthermia, leading to cell death and stimulating the immune system. Favorable results in experiments concerning tumors such as breast cancer [4] and cervical cancer [5] showed their potential to be an adjuvant therapy in the clinic. Clinical trials also showed that PTT-induced hyperthermia has great potential in cancer therapy [6]. Like PTT, photodynamic therapy (PDT) uses photosensitizers (PS) to mediate energy transmission and generate toxic residues, such as reactive oxygen species (ROS). ROS can disrupt DNA and protein structure in cells, leading to organelle destruction and cell death [7]. This process can stimulate the immune system and lead to immunogenic cell death (ICD) [8]. PDT can be used in treating many superficial diseases including but not limited to premalignant conditions and tumors in the skin, digestive system and urinary system [9]. It is an important non-invasive therapy in the clinic. Due to the limited penetration ability of light, sonodynamic therapy (SDT) offers an alternative to PTT and has been demonstrated to have a wide range of applications in different solid tumors such as hepatocarcinoma, glioma and melanoma [10,11]. SDT utilizes sonosensitizers and ultrasound to produce ROS which lead to cancer cell destruction [12,13] {Das, 2024 #181}. SDT plays a vital role in the therapy of deep tumors, as ultrasound can penetrate into deeper tissues. Radiotherapy, chemotherapy and immune therapy are also extensively used to prevent tumor recurrence after surgical removal [14,15,16]. In recent years, immune therapy has flourished as it aims at reactivating patients’ innate immune system against cancer [17,18]. When combined with other therapeutic interventions, immune therapy may provide a silver line against cancer.

The cGAS-STING pathway is considered a potential target for stimulating immune system in patients and achieving a sustained immune response. With intracytoplasmic DNA, cGAS-STING agonist can increase the production of interleukin 6 (IL-6), tumor necrosis factor (TNF) and interferon (IFN) [19] and play a regulatory role in inflammation and tumor treatment [20,21,22]. Deng et al. found that injecting cyclic dinucleotide (CDN) intratumorally after radiotherapy can suppress tumor growth [23]. The cGAS-STING signaling pathway has been extensively studied and adopted for tumor immune therapy [24]. However, administrating a cGAS-STING agonist at a high concentration can lead to significant side effects [25]. It is necessary to find a method to precisely control cGAS-STING activation. Moreover, the effectiveness of cGAS-STING agonists depends on the level of STING expression [26]. When myeloid cells are depleted, the activity of cGAS-STING agonists can be attenuated [27]. In addition, cytokine induced by cGAS-STING can elicit a carcinogenetic impact on epithelial cells [28], and the prolonged overexpression of STING can disrupt the homeostasis in the endoplasmic reticulum (ER), causing ER stress in T cells and leading T-cell death [29]. Recently, Chen et al. reported the use of chitosan hydrogels in photothermal therapy to precisely control the release of a STING agonist in the tumor microenvironment (TME), inducing stable tumor immunity [30]. Yu et al. used a photosensitizer, MHI148, and a STING agonist, 2′3′-cGAMP, to achieve diagnosis and therapeutic effects [31]. Jiang et al. designed a material that used ultrasound to produce singlet oxygen and release a STING agonist to activate tumor immunity [32]. By combining PTT, PDT and SDT, the intrinsic drawbacks of cGAS-STING apoptosis can be mitigated. These synergistic therapies cover a variety of tumors and have the possibility of long-term immunity. The synergistic therapy of non-invasive therapy and immunotherapy is in line with the future direction of tumor therapy. Especially the cGAS-STING pathway holds great promise for immune therapy by mobilizing the patients’ innate immune system to inhibit and eliminate tumors [33]. Some of the STING agonists have reached clinical trials and we list them in Table 1. The reported trials are all in phase I or a combination of phase I and phase II. In five trials, two have been terminated. Three trials are recruiting. The results of E7766 showed dose-limiting toxicities and serious adverse events when the dose escalation came to 600 mcg.

Previous reviews focused on development in photothermal, photodynamic and sonodynamic cancer therapies. For PTT and PDT, most reviews were oriented from a material perspective. Liu’s review provided a general overview of the characteristics of different nanomaterials used in phototherapy and the methods scientists developed to enhance the outcomes [34]. Yang et al. reported on nanomaterials used in SDT and their combination with immune therapies targeting the PD-1/PD-L1 pathway [35]. Moreover, there were limited reviews that concentrated on the application of the cGAS-STING pathway in sonodynamic therapy. Regarding the cGAS-STING pathway, most reviews concentrated on the drug development, different ways to regulate it and its clinical prospects in various diseases [36,37,38]. In this review, we illustrate the combination of the cGAS-STING pathway with photothermal, photodynamic and sonodynamic physical methods in cancer therapy. Relationship and current development of the cGAS-STING pathway to individual cancer therapy method will be discussed in the following sections. We hope this review will provide new insights into the synergistic effects of cGAS-STING agonists and new leads for clinical cancer therapy.

## 2. cGAS-STING Signaling Pathway with Photothermal Therapy

Photothermal therapy (PTT) is a strategy that transfers photoenergy into heat to ablate tumors through hyperthermal effects. Hyperthermia can inhibit tumor repair and facilitate tumor immunity. Song et al. reported that hyperthermia could downregulate oxygenation in tumors, leading to a suppressive environment for the tumor to repair. For normal tissues, the hyperthermia effects were negligible [39,40]. Azocar et al. reported that hyperthermia increased the sensitivity of human natural killer cells [41]. In 2007 and 2021, Ostberg et al. and Pan et al. reported that hyperthermia can promote the anti-tumor effects of NK cells [42,43]. Burd et al. reported that steady heat could cause vascular changes and induce tumor apoptosis [44]. Hyperthermia generated in PTT is mediated by PTAs. PTAs are usually introduced into cells through nanoparticles, as nano PTAs have a higher permeability and can be combined with other therapeutic materials [45]. The surface of PTAs can be modified to control tissue persistence and toxicity [46]. Poursalehi et al. designed chemically modified gold nanoparticles loaded with Doxorubicin, which demonstrated 99% cellular uptake after 3 h [47]. Huilgol et al. reported that the combination of hyperthermia with radiation can significantly improve the response rate of patients and extend median survival [48]. Tang et al. reported using a photosensitive dimer to target the tumor membrane and achieve a synergistic therapy of PTT and PDT [49]. Cell death is followed by the release of tumor-associated antigens (TAAs) and damage-associated molecular patterns (DAMPs). However, TAAs and DAMPs may not be effectively taken up by dendritic cells (DCs) nor induce a T-cell response, as these processes are inhibited in the TME [50].

**Figure 1:**
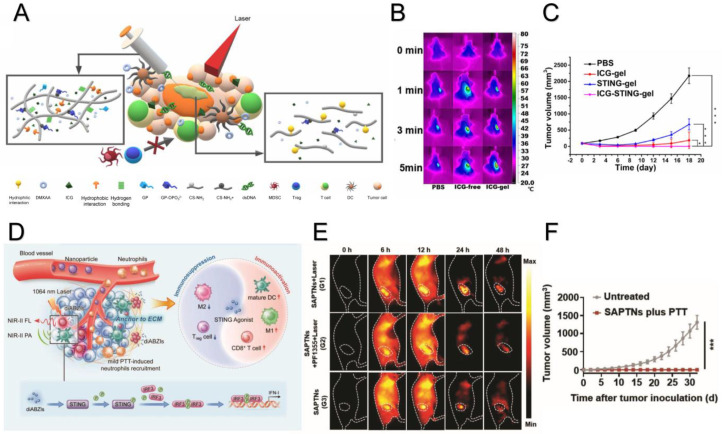
The principles and results of two synergistic therapies of photothermal therapy and STING agonist and their effect on tumor in vivo. (**A**) Schematic illustration of a synergistic therapy of the photothermal transduction agent Indocyanine green (ICG) with the STING agonist DMXAA [30]. (**B**) Temperature development after the injection of ICG of different forms in vivo, the color represents different temperature, and have been shown in the legend [30]. (**C**) Tumor growth in vivo after photothermal therapy and a STING agonist alone and synergistic therapy. * *p* < 0.05 vs. control, *** *p* < 0.001 vs. control [30]. Copyright 2023 American Chemical Society. (**D**) Schematic illustration of a synergistic therapy of the photothermal transduction agent croconaine dye with the STING agonist diABZIs using SAPTNs [60]. (**E**) The images of drug distribution in vivo after the injection of SAPTNs with irradiation alone or with irradiation and the myeloperoxidase inhibitor PF1355, the color represents different temperature and the connection can be seen in the legend [60]. (**F**) Average volume of tumor reinjected in vivo after no treatment in the untreated group and SAPTNs plus PTT group treated by a synergistic therapy of PTT and a STING agonist using SAPTNs. *** *p* < 0.001 vs. control [60]. Copyright 2024 John Wiley and Sons.

Adding immune therapy can enhance the anti-tumor effects of photothermal therapy (PTT) through activating immune system within the TME. The STING pathway is one of the promising options for such a combination. By combining PTT with a STING agonist, the maturation of DCs can be strengthened and cytotoxic T cells can be activated. Cyclic-GMP-AMP (cGAMP) can directly bind to STING and activate the STING pathway [51]. Ishikawa et al. demonstrated that STING is located in the upstream of TBK1/IRF3 and plays an important role in IFN-β expression and innate immune activation [52]. Jiang et al. reported that cGAMP-mediated STING/STAT3 can inhibit the activity, proliferation and invasion of tumor cells and inhibit tumor progression through upregulating IL-2, TNF-α and IFN-γ by cGAMP and downregulating CXCL8, BCL-2 and VEGFA to inhibit angiogenesis [53]. Koshy et al. used liposomes as a delivery vehicle for cGAMP to improve the immune response and achieve immune memory in mice. The injection of liposome-delivered cGAMP could inhibit the growth of metastatic tumors while free drug displayed a limited effect [54]. The theragnostic thermosensitive liposome (PLDD) also demonstrated great potential. Long et al. developed a theragnostic thermosensitive liposome (PLDD) using a D-A-D conjugated oligomer (DTTB) and 5,6-dimethylxanthenone-4-acetic acid (DMXAA)Long [55]. In this combination, DTTB is a newly developed photothermal agent that has demonstrated a high quantum yield and is being evaluated for the pharmaceutical properties of nanomedicine. The PEGylation of DTTB can dramatically improve its blood circulation time and tumor accumulation [56]. Demonstrated by Corrales et al., DMXAA, as a STING agonist, can lead to tumor regression and stimulate strong anti-tumor immunity [57]. However, it showed a limited effect in clinical trials because it turned out to be agonist to mouse STING but not human STING [58,59]. The combination of the photothermal agent DTTB with the STING agonist DMXAA constituted a nanoplatform. When exposed to second near-infrared (NIR-Ⅱ) fluorescence, it could generate heat and lead to ICD, subsequently releasing DMXAA from the thermosensitive liposome. This approach has shown considerable anti-tumor efficiency and biosafety in tumor therapy [55]. Apart from liposomes, Chen et al. used hydrogel to combine the STING agonist DMXAA and the PTA indocyanine green (ICD) (Figure 1A). This hydrogel improved the intensity of ICD (Figure 1B). Combining with DMXAA further improved the anti-tumor effect (Figure 1C) [30]. Ma et al. used the STING agonist diABZIs and the PTA Croconaine dye IR1024 to develop a nanomedicine named STING agonist-based photo-immuno-thernostic nanomedicine (SAPTN) (Figure 1D). SAPTN could stimulate the tumor compared to normal tissue (Figure 1E) and lead to sustained anti-tumor immunity when rechallenged by the same tumor cells (Figure 1F) [60]. In addition, metal ions are reported to stimulate the cGAS-STING pathway; for instance, Mn^2+^ has been demonstrated to activate the immune system through this pathway. Wang’s team found that Mn^2+^ can enhance the sensitivity of cGAS, thereby improving the ability to respond to dsDNA in the cytoplasm. Their study showed that even at low concentration of dsDNA, Mn^2+^ could promote the synthesis of the second messenger cGAMP and enhance the affinity between cGAMP and STING [61]. Mn^2+^ and MoO_4_^2+^ were reported to form a nanoparticle named MMP NDs. MMP NDs could induce tumor cell ferroptosis directly or through reducing the glutathione accumulated in tumor cells as well as activating the cGAS-STING pathway. Furthermore, MMP NDs could stimulate IFN-γ secretion by CD8^+^ T cells and inhibit the expression of GPX4 which promotes ferroptosis [62]. Lin et al. developed a polydopamine-manganese-based nanomaterial [63]. In this report, polydopamine acts as a photothermal agent, and Mn^2+^ can be released with glutathione to produce hydroxyl radicals (·OH) and stimulate the cGAS-STING pathway. This results in the suppression of 86.7% of tumor cells and the production of more cytotoxic T cells compared to the negative immune regulator Treg cells. Moreover, in the experiments of Xia et al. and Zheng et al., another PTA—Prussian blue—was combined with manganese and proved to be effective in colon and breast tumor models [64,65]. These experiments represent that the combination of PTT and STING agonists is a potential strategy for future cancer treatment. Synergistic therapies of photothermal therapy and cGAS-STING agonists in recent studies are listed Table 2 [30,55,56,57,60,63,64,65,66,67,68,69,70,71].

Before photothermal therapy (PTT) can be used clinically, several obstacles must be addressed. First, light attenuation restricts the usage of PTT, especially for internal tumors, where the therapeutic effect of PPT is limited. Additionally, the photosensitizers’ pharmacologic processes, for example, toxicity or metabolism, still require further study. Third, Cherukula et al. reported that the effect of PTT might be limited to 7 days, accompanied by side effects of increased immune tolerance in the tumor microenvironment. But they also found possible therapeutic targets to solve this question [72]. In the experiment of Yue et al., they use an agent named TMP195 to repolarize immunosuppressive tumor-associated macrophages, which also provides a novel way to solve this question [73].

## 3. cGAS-STING Signaling Pathway with Photodynamic Therapy

Photodynamic therapy (PDT) has been clinically approved for over 200 years, commonly used for superficial cancers and in situ cancers such as esophageal cancer [74], skin cancer [75] and gynecologic malignant diseases [76]. For instance, Barrette’s esophagus (BE) is known to progress to high-grade dysplasia (HGD) and adenocarcinoma [77]. In Japan, PTT is applied for esophageal squamous cell carcinoma (ESCC) treatment. The American College of Gastroenterology strongly recommends ablative therapy for residual BE in patients with EMR specimens demonstrating HGD or intramucosal carcinoma. A randomized phase III trial held by 30 centers and 485 patients reported that PDT, when combined with chemotherapy, increases the complete ablation of HGD and reduces the likelihood of adenocarcinoma development [78]. PDT utilizes a specific wavelength of light to generate toxic production through photosensitizers (PS) with tolerable pain. Two pathways contribute to cytotoxicity: the Type I pathway directly transfers electrons from the PS to the oxygen molecule, producing superoxide anions such as free radicals and reactive oxygen species; the Type II pathway involves energy transfer from the PS for singlet oxygen generation [79,80]. Singlet oxygen can penetrate cellular membranes, disrupting protein and DNA. It can also generate damage-associated molecular patterns and mediate the immune response [81]. PDT primarily affects tumor cells, vasculature and the immune system [82]. Shi et al. reported the use of a light-emitting diode for convenient, cost-effective and accurate PDT in gastrointestinal cancer treatment. Their point LED-PDT can offer sufficient light density and induce tumor apoptosis and necrosis [83]. Zhang et al. reported a nano delivery system named R6RGD-CMβCD-se-se-Ce6/LND (RCC/LND NPS), comprising the photosensitizer chlorin e6 (Ce6) and the chemotherapeutic lonidamine (LND). This nanomaterial can disrupt the tumor extracellular matrix (ECM), weaken anoikis resistance in triple-negative breast cancer and activate apoptotic pathways [84]. After injection, the developed photosensitizers accumulate in vasculature tissue [85]. The PS vasculature accumulation and damage can lead to an oxygen-deficient environment, inhibiting the growth of the tumor. The photodynamic reaction can ablate the tumor as well as strengthen tumor immunity [86]. This process includes innate immunity and adaptive immunity. Immune cells, for example, neutrophils, macrophages, NK cells, dendritic cells and T cells, are involved in the immune response [87]. The destruction of tumor tissues creates an inflammatory environment and releases cytokines, leading to dendritic cell (DC) accumulation and maturation. Mature DCs then phagocytose tumor cells, return to lymph nodes, present antigens to CD8^+^ T cells and activate T-cell migration to the tumor [88].

**Figure 2:**
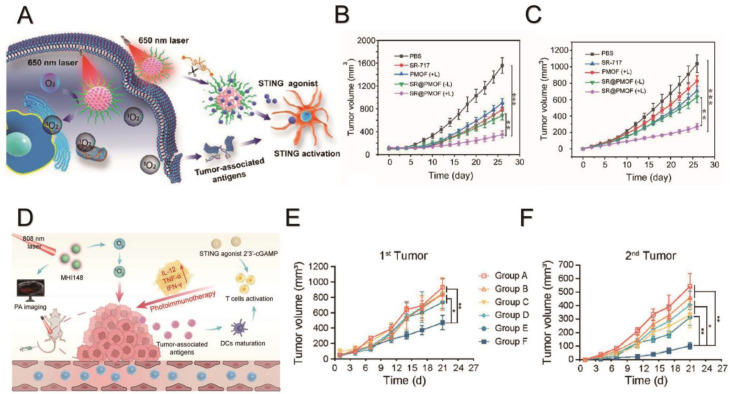
The principle and result of two synergistic therapies of photodynamic therapy and STING agonist on primary tumors and distant tumors in vivo. (**A**) Schematic illustration of a synergistic therapy of the photosensitizer Meso-tetra(carboxyphenyl) porphyrin (TCPP) and STING agonist SR-717 [96]. Volume growth of a primary tumor (**B**) and distant tumor (**C**) when treated with a photodynamic material polymeric metal−organic framework (PMOF) and SR-717 alone and a synergistic therapy of PDT and a STING agonist by using a nanoparticles called SR@PMOF combining PMOF and a STING agonist (SR-717), without light irradiation (-L) or with light irradiation (+L). ** *p* < 0.01 vs. control, *** *p* < 0.001 vs. control [96]. Copyright 2023 American Chemical Society. (**D**) Schematic illustration of a synergistic therapy of a nanoparticle named GM@P, consisting of a hydrophobic shell encapsulating the photosensitizer MHI148 and the STING agonist 2′3′-cGAMP [31]. Tumor growth of primary tumors (**E**) and distant tumors (**F**) after photothermal therapy using a nanoparticle with MHI148 (M@P) alone without irradiation (Group B: M@P), with irradiation (Group D: M@P + light irradiation), with a free STING agonist and irradiation (group E: M@P + light irradiation + 2′3′-cGAMP), a STING agonist alone (Group C: 2′3′-cGAMP), and a synergistic therapy of GM@P (Group F: GM@P + light irradiation) and a control group (Group A). * *p* < 0.05 vs. control, ** *p* < 0.01 vs. control [31]. Copyright 2024 American Chemical Society.

Studies have found that a synergistic therapy of photodynamic therapy (PDT) and a STING agonist is viable. The cGAS-STING signaling pathway, activated during DNA damage from ultraviolet irradiation or cisplatin treatment, induces cell apoptosis and is associated with inflammation and cell senescence [89,90]. Jiang et al. further illuminated the connection between cGAS and cell apoptosis, showing that cGAS inhibits the DNA damage repair process. After DNA damage, cGAS is recruited to double-strand breaks, interacting with PARP1 to hinder the formation of the PARP1–Timeless complex. This inhibits homologous recombination, promotes tumor occurrence, accelerates genomic instability and micronucleus formation and eventually leads to cell death [32,91]. Since PDT can cause DNA damage, adding a STING activator can promote apoptosis in tumor cells. ADU-S100 is a potent synthetic cyclic dinucleotide STING agonist [92]. But a recent study showed its limited clinical effect [93]. Hao et al. reported that using PDT and the STING agonist ADU-S100 can amplify the immune reaction then obtain a systemic immune response and immune memory [94]. Increasing antigen presentation and the repolarization of bone marrow-derived macrophages from the M1 to M2 phenotype have been observed. Both in vivo and in vitro experiments show that this combination can hugely increase the anti-tumor effects compared to monotherapy and demonstrates tumor resistance when rechallenged with tumor cells. SR-717 is a non-nucleotide STING agonist mimicking the structure of cGAMP and can induce a conformation change of STING to activate it [95]. Zhou et al. developed a nanoparticle using a polymeric metal−organic framework (PMOF) containing the photosensitizer Meso-tetra(carboxyphenyl) porphyrin (TCPP). The formulated nanoparticle SR@PMOF combined PMOF and the STING agonist SR-717. After irradiation, ^1^O_2_ produced by PDT can destroy the structure and release SR-717 into the tumor (Figure 2A). The synergistic effect of photodynamic therapy and a STING agonist can strengthen antigen presentation and the infiltration of CD8^+^ T cells and suppress the growth of primary tumors (Figure 2B) and distant tumors (Figure 2C) [96]. Yu et al. further demonstrated that a synergistic therapy of photodynamic therapy and a STING agonist can promote an inflammatory response and tumor suppression (Figure 2D–F) [31]. In Table 2, we list the agents used in the synergistic therapy of photodynamic therapy and a cGAS-STING agonist in recent studies [31,90,93,94,95,96,97,98,99].

Although photodynamic therapy (PDT) has been used in the clinic for a long time, it is still not widely utilized. Issues still needs to be handled. As oxygen is important in the therapy, the destruction of the vasculature can cause oxygen-deficiency in tumor tissues and influence the therapeutic effect [85]. In addition, in clinical settings, adverse effects of PDT are reported in the majority of people [78].

## 4. cGAS-STING Signaling Pathway with Sonodynamic Therapy

Ultrasound is widely used in the clinic for diagnosis and therapy due to its excellent tissue penetration and ability to accumulate sufficient energy for thermal effects [100]. For tumor treatment, sonodynamic therapy (SDT) has more advantages over PDT as light has a limited penetration for deeper tissue. SDT can be categorized based on the intensity of the ultrasound. Low-intensity ultrasound, for instance, can be employed in physiotherapy to heat specific structures. Low-intensity ultrasound (0.51 W/cm^2^, 1.0 MHz, 10 min) was reported for use on mouse squamous cell carcinoma (SCC), showing an anti-tumor effect [101]. High-intensity ultrasound (ranging from 10^3^ to 10^4^ W cm^−2^ with frequencies from 0.5 to 10.0 MHz) of 1.5 MHz is most commonly used for cancer treatment. Similar to photosensitizers, sonosensitizers can be activated by ultrasound to produce reactive intermediates that release free radicals. In addition, cavitation is also the main theory regarded as the mechanism of SDT. In the process of cavitation, bubbles are created when irradiated by ultrasound. These bubbles can expand and collapse at different phases. When these bubbles collapse, the temperature and pressure increase and oxidants are formed [102]. The resulting oxidants can induce the generation of ROS and the leakage of mitochondrial DNA in cytosol, damaging tumor tissues and vasculature [103]. In addition, cavitation can produce shock waves and shear stress to directly cause mechanical damage to cancer cells [104]. Wood et al. reported that low-intensity SDT could cause an antivascular effect in tumor tissues, creating an ischemic environment and leading to cell death [105,106]. SDT has been used to ablate tumors at a high temperature from 60 °C to about 95 °C in prostate cancer, hepatic cancer and esophageal cancer [105]. SDT uses sonosensitizers that harness ultrasound energy to eliminate the biofilm of cancer cells. These sonosensitizers also possess imaging capabilities, aiding in the precise delineation of target areas [107]. Wang et al. combined SDT with photodynamic therapy in a clinical trial for patients with advanced breast cancer, observing cancer degradation [108].

**Figure 3:**
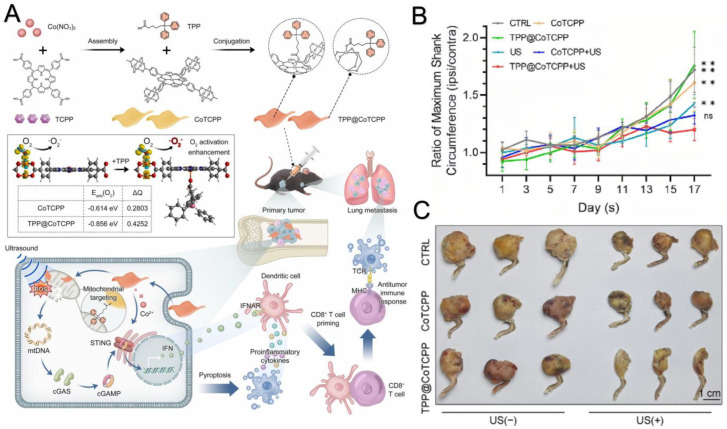
The principle of a synergistic therapy of sonodynamic therapy and a cGAS-STING agonist using a material consisting of triphenyl phosphonium (TPP) and sonodynamic cobalt organic frame-work nanosheets (TPP@CoTCPP) and some experimental results. (**A**) Graphic illustration of TPP@CoTCPP [111]. (**B**) Comparison of the volume of ipsilateral and contralateral tumor in vivo in different groups treated by sonodynamic therapy and a STING agonist alone and a synergistic therapy of sonodynamic therapy and a cGAS-STING agonist. ns indicates not significant, ** *p* < 0.01 vs. control [111]. (**C**) Tumor volume of sonodynamic therapy alone and synergistic therapy [111]. Copyright 2023 Elsevier.

In sonodynamic therapy (SDT), cell apoptosis triggers the release of tumor-derived DNA. However, the immune system in TME is suppressive. This issue can be addressed by synergistic therapy with cGAS-STING agonists. cGAMP and other STING agonists can directly activate DC and enhance the presentation of related antigens to CD8^+^ T cells in vitro, thus promoting the activation of CD8^+^ T cells and their killing effect on tumor cells. cGAMP and other STING agonists can directly activate DCs and enhance the presentation of related antigens to CD8^+^ T cells in vitro [54], thus promoting the activation of CD8^+^ T cells and their killing effect on tumor cells. The use of a STING agonist can enhance the activation, cytotoxicity and anti-tumor effects of natural killer (NK) cells independently of CD8^+^ T cells, thus improving the clearance of tumors resistant to CD8+ T cells [109]. Using a STING agonist can revive the silenced immune system in “cold” tumors, turning them into “hot” tumors by inducing the infiltration of CD8^+^ T cells to activate anti-tumor immunity, thereby improving tumor clearance [110]. Reported by Lei et al., a cobalt-based nanoagonist was combined with the mitochondria-targeting ligand triphenyl phosphonium (TPP). TPP can target mitochondria and induce the leakage of mitochondrial DNA with the involvement of SDT. In the presence of cobalt, STING activation is more effective. And controllable activation of the immune system can be achieved, even in bone and metastatic tumors [111] (Figure 3). Jiang et al. and Yu et al. used a semiconducting polymer to combine with an orally available agonist agent MSA-2 [112,113,114]. Yu et al. designed polymeric STING pro-agonists to target the tumor microenvironment with elevated glutathione expression and the improved release of MSA-2 [113]. Lu et al. use the clinically approved sonosensitizer and STING agonist SR-717 [95,115]. An anaerobic microorganism of *Bifidobacteria Longum* (BiL) was designed, demonstrated sono-sensitivity in anaerobic environments and was named HMME@BiL. HMME@BiL demonstrated high efficacy and selectivity and good biocompatibility against malignant tumors [116]. Tian et al. used zinc oxide, zinc ions as sonosensitizer and a STING agonist (PZnO@DOX) [117,118,119]. PZnO@DOX was proved to modulate the immunogenic cell death induced by chemotherapy [119]. We summarize the synergistic therapy of sonodynamic therapy and cGAS-STING agonists of recent studies in Table 2 [95,112,113,114,115,116,117,118,119,120].

While scientists have reported cases using sonodynamic therapy (SDT) with other therapies, a systemic analysis of the therapeutic effects of SDT on cancer and the immune system is still required. Pain could occur in patients when energy accumulates in deep organs especially in the bones [108]. Moreover, no entirely suitable sonosensitizer has been developed for clinical use.

**Table 2:** Overview of synergistic therapy of PDT, PTT and SDT with cGAS-STING agonists.

Therapy			References
Photothermal Therapy	Photothermal Transduction Agents	STING agonist	
	Indocyanine green	DMXAA	[30,57,66]
	Croconaine dye IR1024	DiABZIs	[60,67,68]
	DTTB	DMXAA	[55,56,57]
	Prussian blue	Manganese	[64,65,69,70]
	Polydopamine	Manganese	[63,70,71]
Photodynamic Therapy	Photosensitizers	STING agonist	
	Verteporfin	ADU-S100	[93,94,97]
	Meso-tetra(carboxyphenyl) porphyrin (TCPP)	SR-717	[95,96,98]
	MHI148	2′3′-cGAMP	[31,90,99]
Sonodynamic Therapy	Sonosensitizers	STING agonist	
	Semiconducting polymer	MSA-2	[112,113,114,120]
	Hematoporphyrin monomethyl ether	SR-717	[95,115,116]
	Zinc oxide	Zinc ions	[117,118,119]

## 5. Summary and Outlook

Recent progress has emerged with promising solutions to advance photothermal, photodynamic and sonodynamic cancer therapy. Addressing the challenges of limited light penetration and oxygen deficiency in the tumor environment, Tian et al. utilized a singlet oxygen battery (SOB) to release ROS independently of oxygen and light and to control the release within the tumor [121]. Shaw et al. also reported that by modulating the concentration and distribution of nanoparticles and the range of irradiation, thermal damage can be achieved for tumors at depths of up to 9 mm [122]. But we still have a long way to go in clinical cancer therapy, particularly in the aspects of therapeutic efficacy, material safety, minimal side effects, etc. In addition, some agents in PTT, PDT and SDT have reached clinical trials, and we have listed some of them in Table 3. For photothermal therapy, only one reported trial has reached phase III. For photothermal therapy, the safety issue still needs more clinical confirmation. For photodynamic therapy, four of them have reached phase II and one has reached phase IV. Three trials are recruiting and four have not started to recruit. For sonodynamic therapy, three trials have reached phase I and two trials have reached phase II. Three out of five clinic trials are under active recruiting.

We hope this review will provide a glimpse into a synergistic effect with cGAS-STING agonists and their potential in clinic application. Combining photothermal, photodynamic and sonodynamic therapeutic methods with cGAS-STING agonists can help to overcome the blockade of the immune-suppressive environment within tumors. In turn, those therapeutic methods can strengthen the stimulation of the cGAS-STING pathway as they can induce cell death and the release of tumor-associated antigen. In addition, side effects caused by cGAS-STING agonists can be accurately controlled by synergistic application of these methods. These three kinds of synergistic therapy can be applied in various tumors and lead to an efficient, persistent systemic anti-tumor effect compared to monotherapy. They shine a light on a new direction of clinical application along with chances and challenges. In conclusion, the combination of photothermal, photodynamic and sonodynamic therapeutic methods with cGAS-STING agonists could offer more precise and safe approaches with broad future prospects for cancer therapy.

## Figures and Tables

**Table 1:** Summary of clinical trials of a STING agonist published on the NIH website (https://clinicaltrials.gov/ by 13 July 2024).

Clinical Trials ID	Study Start	Drug	Sponsor	Study Status	Study Phase
NCT04144140	2020-02	E7766	Eisai Inc. (Tokyo, Japan)	Terminated	Phase I/Ib
NCT04609579	2020-11	SNX281	Stingthera, Inc. (Boston, MA, USA)	Terminated	Phase I
NCT05070247	2022-04	TAK-500	Takeda (Tokyo, Japan)	Recruiting	Phase I/II
NCT05387928	2022-06	KL340399	Sichuan Kelun Pharmaceutical Research Institute Co., Ltd. (Chengdu, China)	Recruiting	Phase I
NCT06021626	2023-08	CRD3874-SI	Memorial Sloan Kettering Cancer Center (New York, NY, USA)	Recruiting	Phase I

**Table 3:** Summary of photothermal, photodynamic and sonodynamic therapies in clinical trials published on the NIH website (https://clinicaltrials.gov/ by 13 July 2024).

Therapy	Clinical Trials ID	Study Start	Report Title	Sponsor	Study Status	Study Phase
Photothermal Therapy	NCT01679470	2012-10	Efficacy Study of AuroLase Therapy in Subjects with Primary and/or Metastatic Lung Tumors	Nanospectra Biosciences, Inc. (Houston, TX, USA)	Terminated	Not Applicable
	NCT03202446	2016-06	Randomized Clinical Trial Evaluating the Use of the Laser-Assisted Immunotherapy (LIT/inCVAX) in Advanced Breast Cancer	Eske Corporation S.A.C (Lima, Peru)	Terminated	Phase III
Photodynamic therapy	NCT05386056	2022-12	Pembrolizumab and Photodynamic Therapy in Previously Treated Metastatic Esophageal Squamous Cell Carcinoma	Peking University (Beijing, China)	Not Yet Recruiting	Phase II
	NCT05551299	2023-02	Treatment of Non-resectable Bile Duct Cancer with Radiofrequency Ablation or Photodynamic Therapy (CARP)	University of Leipzig (Leipzig, Germany)	Recruiting	Phase IV
	NCT05736406	2024-02	A Dose-escalation Clinical Study of Intraoperative Photodynamic Therapy of Glioblastoma	Hemerion Therapeutics (Villeneuve d’Ascq, France)	Recruiting	Phase I/II
	NCT05374915	2024-02	Efficacy and Safety Study of REM-001 Photodynamic Therapy for Treatment of Cutaneous Metastatic Breast Cancer (CMBC)	Kintara Therapeutics, Inc. (San Diego, CA, USA)	Recruiting	Phase II
	NCT06381154	2024-06	Photoradiation with Verteporfin to Facilitate Immunologic Activity of Pembrolizumab in Unresectable, Locally Advance or Metastatic Pancreatic Cancer	Mayo Clinic (Scottsdale, AZ, USA)	Not Yet Recruiting	Phase II
	NCT06306638	2024-07	Interstitial Photodynamic Therapy Following Palliative Radiotherapy in Treating Patients with Inoperable Malignant Central Airway Obstruction	Roswell Park Cancer Institute (Buffalo, NY, USA)	Not Yet Recruiting	Phase I/II
	NCT06437288	2024-07	Hematoporphyrin Photodynamic Therapy for Esophageal Cancer	Sun Yat-sen University (Guangzhou, China)	Not Yet Recruiting	Phase II
Sonodynamic therapy	NCT04559685	2021-03	Study of Sonodynamic Therapy in Participants with Recurrent High-Grade Glioma	Nader Sanai (Phoenix, AZ, USA)	Recruiting	Early Phase I
	NCT05362409	2022-06	Study to Evaluate 5-ALA Combined with CV01 Delivery of Ultrasound in Recurrent High Grade Glioma	Alpheus Medical, Inc. (Chanhassen, MN, USA)	Active, Not Recruiting	Phase I
	NCT05123534	2022-08	A Phase 2 Study of Sonodynamic Therapy Using SONALA-001 and Exablate 4000 Type 2.0 in Patients With DIPG	SonALAsense, Inc. (Berkeley, CA, USA)	Recruiting	Phase II
	NCT04845919	2023-02	Sonodynamic Therapy with ExAblate System in Glioblastoma Patients (Sonic ALA)	Fondazione I.R.C.C.S. Istituto Neurologico Carlo Besta (Milan, Italy)	Not Yet Recruiting	Phase II
	NCT06039709	2024-01	Sonodynamic Therapy in Patients with Recurrent GBM (GBM 001)	Shayan Moosa, MD (Charlottesville, VA, USA)	Recruiting	Phase I
